# Growth Hormone Secretagogues Protect Mouse Cardiomyocytes from *in vitro* Ischemia/Reperfusion Injury through Regulation of Intracellular Calcium

**DOI:** 10.1371/journal.pone.0035265

**Published:** 2012-04-06

**Authors:** Yi Ma, Lin Zhang, Joshua N. Edwards, Bradley S. Launikonis, Chen Chen

**Affiliations:** 1 School of Biomedical Sciences, University of Queensland, Brisbane, Queensland, Australia; 2 School of Biological Sciences, University of Auckland, Auckland, New Zealand; University of Florida, United States of America

## Abstract

**Background:**

Ischemic heart disease is a leading cause of mortality. To study this disease, ischemia/reperfusion (I/R) models are widely used to mimic the process of transient blockage and subsequent recovery of cardiac coronary blood supply. We aimed to determine whether the presence of the growth hormone secretagogues, ghrelin and hexarelin, would protect/improve the function of heart from I/R injury and to examine the underlying mechanisms.

**Methodology/Principal Findings:**

Isolated hearts from adult male mice underwent 20 min global ischemia and 30 min reperfusion using a Langendorff apparatus. Ghrelin (10 nM) or hexarelin (1 nM) was introduced into the perfusion system either 10 min before or after ischemia, termed pre- and post-treatments. In freshly isolated cardiomyocytes from these hearts, single cell shortening, intracellular calcium ([Ca^2+^]_i_) transients and caffeine-releasable sarcoplasmic reticulum (SR) Ca^2+^ were measured. In addition, RT-PCR and Western blots were used to examine the expression level of GHS receptor type 1a (GHS-R1a), and phosphorylated phospholamban (p-PLB), respectively. Ghrelin and hexarelin pre- or post-treatments prevented the significant reduction in the cell shortening, [Ca^2+^]_i_ transient amplitude and caffeine-releasable SR Ca^2+^ content after I/R through recovery of p-PLB. GHS-R1a antagonists, [D-Lys3]-GHRP-6 (200 nM) and BIM28163 (100 nM), completely blocked the effects of GHS on both cell shortening and [Ca^2+^]_i_ transients.

**Conclusion/Significance:**

Through activation of GHS-R1a, ghrelin and hexarelin produced a positive inotropic effect on ischemic cardiomyocytes and protected them from I/R injury probably by protecting or recovering p-PLB (and therefore SR Ca^2+^ content) to allow the maintenance or recovery of normal cardiac contractility. These observations provide supporting evidence for the potential therapeutic application of ghrelin and hexarelin in patients with cardiac I/R injury.

## Introduction

Cardiac ischemia is one of the leading causes of mortality in the world. It is caused by a temporary interruption of blood flow in the arteries of the heart [1]. The primary clinical therapeutic strategy for treatment of cardiac ischemia is reperfusion. However, reperfusion can cause additional injury to the heart [1], [2]. Recovery of cardiac function following ischemia is critically dependent on the time spent under ischemic conditions and reperfusion [3].

*In vitro* global and *in vivo* regional ischemia/reperfusion (I/R) models have been developed to examine experimentally cardiac ischemia and subsequent reperfusion of the ischemic heart. The *in vivo* regional I/R model mimics atherosclerosis by ligating the left anterior descending coronary artery. The global I/R model for *in vitro* study blocks all perfusion of the heart for a given period. The latter can be easily implemented and affects a larger area with a less variability among different regions [4]. It is used to mimic the process of cardiac arrest and cardiac surgery [5]. This model is also more appropriate for obtaining isolated cells which have been through similar ischemia conditions without the regional differences often observed in regional I/R models.

In the past 50 years, great progress has been made to clarify the metabolic changes that occur following I/R [1], [2], [6], [7]. During ischemia, depletion of oxygen and ATP inhibits SR Ca^2+^ ATPase (SERCA2a) and Na^+^-K^+^ ATPase activities. This results in an accumulation of intracellular Ca^2+^ ([Ca^2+^]_i_) and Na^+^ ([Na^+^]_i_) [1], [2], [6], [7]. The subsequent reintroduction of oxygen during reperfusion leads to the generation of large amounts of reactive oxygen species (ROS), causing increased oxidative stress and subsequent damage to the plasma and SR membranes resulting in further increases in [Ca^2+^]_i_. The combined effects of ROS and [Ca^2+^]_i_ overload also favor the opening of the mitochondrial permeability transition pore (mPTP), which induces cardiomyocyte apoptosis and necrosis [1], [2], [6], [7].

Ghrelin is a 28 amino acid peptide produced in the stomach and is an endogenous ligand of the growth hormone secretagogue (GHS) receptor type 1a (GHS-R1a) [8]. A synthetic analogue of ghrelin, hexarelin, also binds and activates the GHS-R1a [9], [10], [11]. Ghrelin mainly exists in the pituitary and gastrointestinal system [8], [12], while the distribution of its receptor GHS-R1a is ubiquitous and has been confirmed in the myocardium [12], [13]. Although ghrelin may bind to receptors other than GHS-R1a [14], [15], [16], its main target is GHS-R1a.

Previous studies have confirmed the protective effects of GHS on whole heart function after I/R. Administration of ghrelin *in vitro* to I/R rat hearts was shown to reduce the infarct size [17], and enhance cardiac function [18] through the activation of PKC [17]. These effects are likely initiated by the binding of ghrelin to its receptor, GHS-R1a [18]. Further studies in rats pre-treated with GHS for 7 days *in vivo* prior to *in vitro* I/R injury showed an improvement in cardiac function [10] and attenuation of myocardial injury and apoptosis through the inhibition of endoplasmic reticulum (ER) stress [19]. Similarly, hexarelin has also been shown to play a cardioprotective role in I/R hearts from rodents [10], [17], [20].

As discussed above, whole heart functional studies employing the *in vivo* and *in vitro* I/R models have revealed some potential mechanisms of the cardioprotective effects of GHS. Detailed cellular and molecular pathways employed by GHS through activation of GHS-R1a after cardiac I/R remain elusive. Since [Ca^2+^]_i_ plays a critical role in cardiomyocyte contraction and I/R injury, in this study we investigated the alterations in and regulation of [Ca^2+^]_i_ homeostasis in isolated mouse cardiomyocytes with or without I/R and GHS treatment.

## Methods

### Animals and Chemicals

All experiments conformed to the Guide for the Care and Use of Laboratory Animals published by the US National Institutes of Health (NIH Publication No.85–23, revised 1996), and the protocol was approved by the Animal Ethics Committee of the University of Queensland (AEC # SBMS/814/07/NHMRC). All surgeries were performed under sodium pentobarbital anesthesia, and all efforts were made to minimize suffering.

Human ghrelin was obtained from Auspep (Parkville, Australia). Hexarelin was obtained from GL Biochem (Shanghai, China). Pentobarbital sodium was purchased from Virbac Pty Ltd (Australia). Heparin sodium salt was purchased from Sigma Aldrich (St. Louis, MO, USA). Fura 2-AM was purchased from Invitrogen (Eugene, Oregon, USA). [D-Lys3]GHRP-6 was purchased from Anaspec Inc. (San Jose, CA). BIM28163 was kindly provided by Michael D. Culler (Ipsen Pty Ltd, Australia). Other chemicals for recording solutions were purchased from Sigma (St. Louis, MO, USA).

### *In vitro* Ischemia/Reperfusion Model and Preparation of Ventricular Myocytes

Adult male C57/B1 mice (7 to 9 weeks old) weighing between 34 g and 36 g were anesthetized with sodium pentobarbitone (40 mg/kg, ip) containing heparin (500 Units, ip). The heart was rapidly excised, cannulated and perfused retrogradely via the aorta with Tyrode solution at 3 ml/min on a Langendorff perfusion apparatus (composition in mM: 10 HEPES, 143 NaCl, 5.4 KCl, 0.5 MgCl_2_, 10 Glucose, 20 Taurine, 1.5 CaCl_2_; pH 7.4; bubbled with 100% O_2_ at 37°C).

The times for stabilization, ischemia and reperfusion were similar to previous studies [3], and are generally considered the most appropriate for functional studies using an *in vitro* I/R model. After 20 min of stabilization, the heart was subjected to 20 min of no-flow global ischemia followed by 30 min of reperfusion. Control hearts were continuously perfused for 70 min. Ghrelin (10 nM) or hexarelin (1 nM) was administered in the perfusion solution before or after ischemia for 10 min [13], termed GHS pre-treatment and post-treatment respectively. In some experiments, the GHS-R1a antagonist [D-Lys3]-GHRP-6 (200 nM) or BIM28163 (100 nM) was introduced into the perfusion system 5 min before the onset of ischemia and remained present throughout (15 min in total).

Following perfusion, cardiomyocytes were isolated from the left ventricle of each heart with Tyrode solution containing 100 µM CaCl_2_, 0.6 mg/ml collagenase Type II (Worthington, NJ, USA) and 0.1 mg/ml proteinase type XXIV (Sigma, MO, USA). The Ca^2+^ level was gradually increased to 1.5 mM over 30 min. The yield of this isolation was usually around 60 – 70%. Only cardiomyocytes that were quiescent with a rod shape, sharp edges and clear striations were used in this investigation. At least 3 hearts were used in each group.

### Measurement of Sarcomere Shortening

Sarcomere shortening was measured as previously described [21]. In brief, cardiomyocytes were electrically stimulated at 0.5 Hz until contractions became uniform. Following this, 10 – 20 consecutive contractions were recorded. The percentage of sarcomere shortening, time-to-peak shortening and time-to-90% relaxation were determined by IonWizard software (IonOptix Corporation, MA).

### Measurement of Intracellular Ca^2+^ Transients and SR Ca^2+^ Content

The isolated and Ca^2+^-tolerant cardiomyocytes were loaded with 5 µM Fura-2 AM (Invitrogen, CA, USA) for 10 min at room temperature. Cardiomyocytes were observed through a Nikon fluor ×40 oil immersion objective and positioned for recording of Fura-2 fluorescence signals. During field stimulation at 0.5 Hz, cytoplasmic Fura-2 was excited by an IonOptix Hyperswitch dual-excitation light source (IonOptix Corporation, MA) at 340 and 380 nm and emitted light collected in a photomultiplier tube. [Ca^2+^]_i_ concentration was inferred from the ratio (R) of the intensity of the emitted fluorescence signals. Amplitude, time-to-peak, time-to-90% decay, and rate of rise (dR/dt) of the derived [Ca^2+^]_i_ transients were determined by IonWizard software.

For estimation of SR Ca^2+^ content, cardiomyocytes with cytoplasmic Fura-2 were paced at least 15 times at 0.5 Hz and then stopped. About 30s later, 10 mM caffeine was added to induce SR Ca^2+^ release. The area under the caffeine-induced [Ca^2+^]_i_ transient (area under curve, AUC) and its amplitude were used as a reflection of the SR Ca^2+^ content [22]. Time-to-90% decay of caffeine-induced increase in [Ca^2+^]_i_ was also measured to estimate the Ca^2+^ clearance ability of Na^+^-Ca^2+^ exchanger (NCX).

### RT–PCR

Total cellular RNA was extracted from left ventricle, septum and right ventricle of mouse hearts using a TRIzol Plus RNA Purification kit (Invitrogen, CA, USA). Single-stranded cDNA was synthesized from 2 µg total RNA with an iScript cDNA Synthesis kit (Bio-Rad Laboratories, CA, USA) following the manufacturer’s instructions.

PCR was performed using JumpStart *Taq* DNA polymerase (Sigma, MO, USA), the cDNA generated above and the corresponding primers for GHS-R1a [23] (Forward: TCATCGATCACAGCCATGT; Reverse: AAGCCAAACTGACCATGT; Tm  =  64°C, 40 cycles). Mouse 18s rRNA was amplified as a control. Following our previous report [13], liver and pituitary were chosen as negative and positive controls respectively for GHS-R1a. PCR products were separated by agarose gel electrophoresis (2%), stained with ethidium bromide and visualized under UV light.

### Western Blotting

Protein expression of the GHS receptor GHS-R1a and the phosphorylated phospholamban (p-PLB)/phospholamban (PLB) that are essential for SERCA2a activity were examined by Western blot analysis according to previous reports [24], [25]. In brief, proteins were extracted from isolated cardiomyocytes (GHS-R1a) or minced mouse left ventricles (p-PLB and PLB) in lysis buffer. Extracted proteins (100 µg) were denatured at 37°C for 30 min (p-PLB and PLB) or 70°C for 10 min (GHS-R1a) in 2× sample buffer, separated on 10–15% SDS-polyacrylamide gels and transferred to nitrocellulose membranes. After blocking, the membrane was incubated with polyclonal rabbit anti- mouse phospholamban (phospho S16,1∶1000; abcam, Cambridge, MA, USA), polyclonal rabbit anti- mouse phospholamban (1∶1000; abcam) or polyclonal goat anti-human GHS-R1a (1∶1000; Santa Cruz Biotechnology, Inc., Santa Cruz, CA, USA) primary antibodies overnight at 4°C before incubation with the corresponding secondary antibodies (1∶5000) and detection with enhanced chemiluminescence (Pierce) according to the manufacturer’s instructions. Mouse GAPDH (1∶2000; Milipore, Billerica, MA, USA) was used as the internal control to allow semi-quantitative densitometry analysis on scanned films using ImageJ software [24].

### Statistical Analysis

All data were expressed as mean ± S.E.M. One-way ANOVA with Tukey *post hoc* test was carried out for multiple comparisons as appropriate. In all comparisons, the differences were considered to be statistically significant at a value of *P* < 0.05.

## Results

### GHS-R1a Expression in Mouse Heart

**Figure 1:**
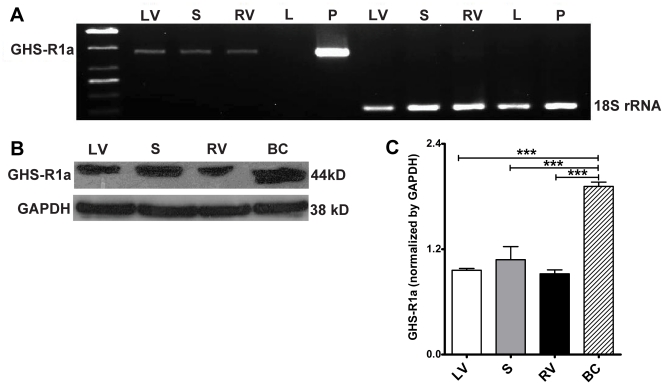
mRNA (A) and protein (B and C) expression of GHS-R1a in mouse heart. (A) Liver (L) and pituitary gland (P) were used as the negative and positive controls respectively. Mouse 18s rRNA was chosen as an internal control. (B) A breast cancer (BC) cell line was used as the positive control. (C) n  =  3 for Western blots, data are shown as means ± S.E.M. and analyzed by One-way ANOVA with *Tukey’s post hoc* test. ***P < 0.001 vs BC group. GHS-R1a mRNA and protein are expressed in the left ventricle (LV), septum (S) and right ventricle (RV) of the mouse heart.

The mRNA and protein expression of GHS-R1a, a GHS receptor in the mouse heart, was examined by RT-PCR and Western blots. As shown in FIG.1, GHS-R1a mRNA and protein is distributed in different regions of mouse heart, including left ventricle, septum and right ventricle. However, the protein expression level was relatively low compared to the breast cancer cell line used as positive control [24].

### Effect of Ghrelin and Hexarelin on Sarcomere Shortening and Intracellular Ca^2+^ Transients after I/R

**Figure 2:**
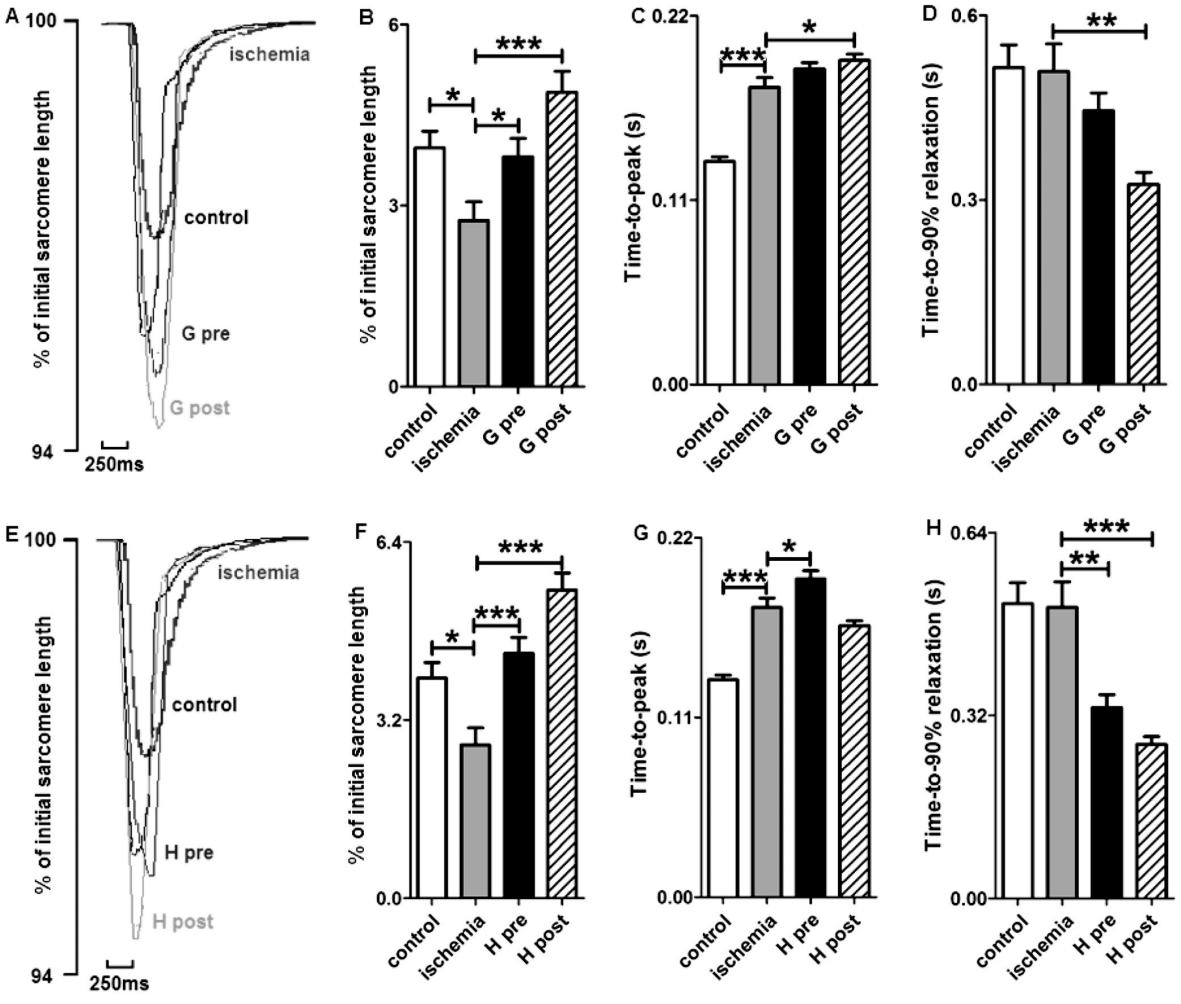
Effects of GHS on the contractile properties of mouse cardiomyocytes exposed to ischemia/reperfusion. (A) and (E) are representative superimposed traces of sarcomere shortening after ghrelin (G) or hexarelin (H) pre-treatment (pre) and post-treatment (post). Both hexarelin and ghrelin improved the reduction in sarcomere shortening (B and F), but not the prolonged time-to-peak shortening (C and G). The time-to-peak shortening was further prolonged in G post- and H pre-treatment groups. The time for relaxation (D and H) was shortened in G post-, H pre- and post-treatment groups. n  =  99, 84, 84, 95, 106 and 100 cells/3 mice in control, ischemia, G pre, G post, H pre and H post groups, respectively. Data are shown as means ± S.E.M. and analyzed by One-way ANOVA with *Tukey’s post hoc* test. *P < 0.05, ** *P* < 0.01, *** *P* < 0.001 vs ischemic group.

**Figure 3:**
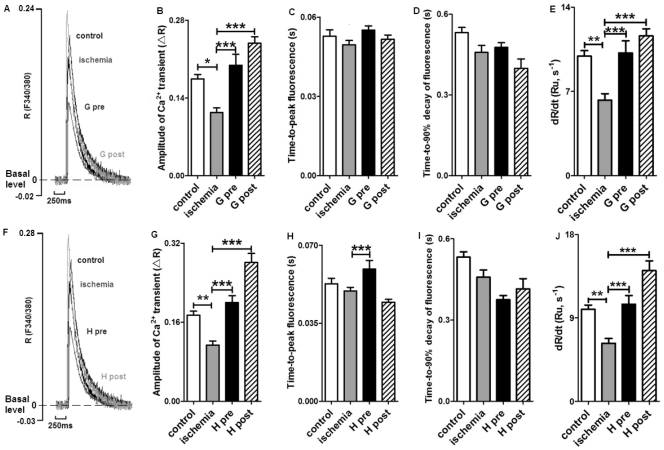
Effects of GHS on [Ca^2+^]_i_ transients in cardiomyocytes exposed to ischemia?reperfusion. (A) and (F) are representative superimposed traces of [Ca^2+^]_i_ transient after ghrelin (G) or hexarelin (H) pre-treatment (pre) and post-treatments (post). R represents the emission fluorescence ratio of fura-2 from excitation at 340 and 380nm, and Ru represents the ratio unit. Both hexarelin and ghrelin improved the reduced amplitude of [Ca^2+^]_i_ transients (B and G). The time-to-peak fluorescence was prolonged in ghrelin and hexarelin pre-treatment (C and H) groups, but the decreased rate of rise of [Ca^2+^]_i_ transients after ischemia was reversed to control level by all H and G pre- and post-treatments (E and J). Ghrelin and hexarelin had no effect on the time-to-90% decay of [Ca^2+^]_i_ transient (D and I). n  =  72, 80, 90, 103, 63 and 66 cells/3 mice in control, ischemia, G pre, G post, H pre and H post groups, respectively. Data are shown as means ± S.E.M. and analyzed by One-way ANOVA with *Tukey’s post hoc* test. *P < 0.05, **P < 0.01, *** *P* < 0.001 vs ischemic group.

To determine the effects of ghrelin and hexarelin on cardiomyocyte function after I/R, cell shortening (FIG. 2A and E) and [Ca^2+^]_i_ transients (FIG. 3A and 3F) were recorded during stimulation at 0.5 Hz.

Sarcomere shortening was expressed as percentage of the resting sarcomere length (FIG. 2). It was found that relative sarcomere shortening was significantly reduced after I/R. However, the presence of 10 nM ghrelin or 1 nM hexarelin during pre- and post-treatments protected cardiomyocytes against this negative effect of I/R (FIG. 2B and F). The amplitude of the corresponding [Ca^2+^]_i_ transients decreased in the I/R group, as determined by the cytoplasmic fura-2 ratio changes shown in FIG. 3. Again, GHS treatment showed protective effects in cardiomyocytes against I/R-induced reductions in [Ca^2+^]_i_ transients (FIG. 3).

Basal [Ca^2+^]_i_ levels were significantly increased after 20 min ischemia (control vs ischemia: 1.63 ± 0.03, n  =  72 cells vs 1.80 ± 0.05, n  =  80 cells; *P <* 0.01), which is consistent with previous reports describing increased cytoplasmic Ca^2+^ and possible Ca^2+^ overload after ischemia [1], [2]. The increased basal [Ca^2+^]_i_ after I/R was not reversed by either ghrelin pre- (1.99 ± 0.04, n  =  90 cells, *P* < 0.05) and post-treatment (2.23 ± 0.05, n  =  103 cells, *P* < 0.001), or hexarelin pre- (2.24 ± 0.05, n  =  63 cells, *P* < 0.001) and post-treatment (2.20 ± 0.06, n  =  66 cells, *P* < 0.001).

GHS treatment also influenced the time course of sarcomere shortening (FIG. 2) and [Ca^2+^]_i_ transients after I/R (FIG. 3). First, ghrelin post-treatment (FIG. 2C) and hexarelin pre-treatment (FIG. 2G) further prolonged the increased time-to-peak shortening following I/R. In addition, the corresponding time-to-peak of [Ca^2+^]_i_ transients in the ischemic group was also delayed by hexarelin pre-treatment (FIG. 3H). Furthermore, the time-to-90% decay of the [Ca^2+^]_i_ transients after GHS treatments (FIG. 3D and I) was similar to that in the ischemic group without GHS treatment, but nearly all GHS treatments except ghrelin pre-treatment (FIG. 2D and H) shortened the time-to-90% relaxation of the force compared to the I/R group. This indicates a lusitropic effect of GHS. As shown in FIG. 3E and J, the decrease in the rising rates of [Ca^2+^]_i_ transients after I/R was also diminished by all GHS treatments, which may indicate the effect of GHS on Ca^2+^ influx through L type Ca^2+^ current (*I*_CaL_), Ca^2+^ release from SR through ryanodine receptors (RyR2s) or both.

### Effect of Ghrelin and Hexarelin on SR Ca^2+^ Content after I/R

**Figure 4:**
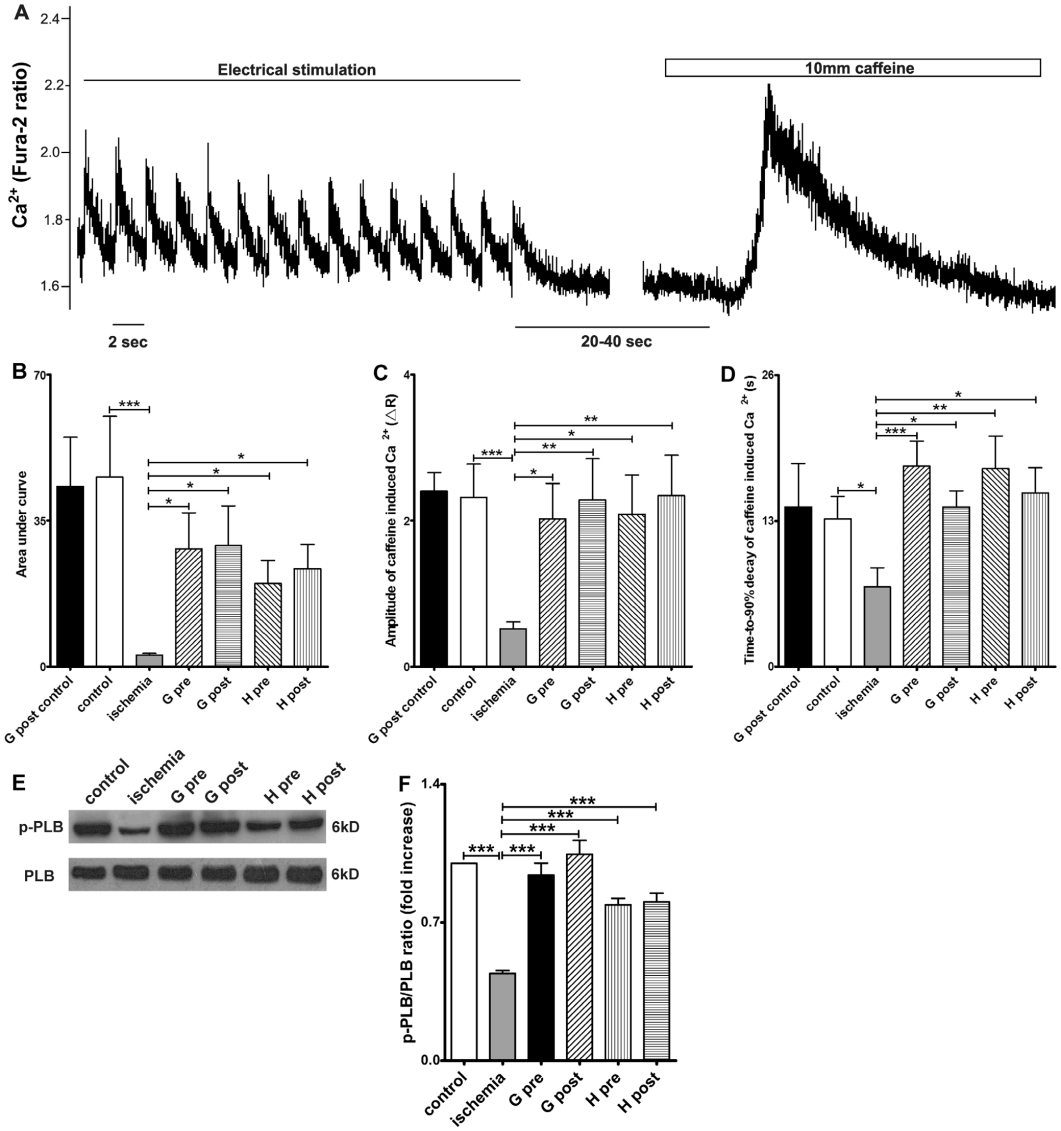
Effects of GHS on sarcoplasmic reticulum (SR) Ca^2+^ content and phospho-phospholamban (p-PLB)/phospholamban (PLB) expression. (A) Illustration of the SR Ca^2+^ content measurement protocol (data from cardiomyocytes exposed to ischemia/reperfusion). R represents the emission fluorescence ratio of fura-2 from excitation at 340 and 380nm. Cardiomyocytes were perfused with Tyrode solution containing 1.5 mM CaCl_2_ and paced at 0.5 Hz for at least 30 s. 10 mM caffeine was then added to induce SR Ca^2+^ release. SR Ca^2+^ content, as determined by both (B) Area under curve and (C) amplitude of the caffeine-induced Ca^2+^ release, significantly decreased after 20 min ischemia compared with the control group. Ghrelin (G) or hexarelin (H) pre-treatment (pre) and post-treatment (post) significantly increased the SR Ca^2+^ content after 20 min ischemia (B and C), but introduction of ghrelin into the perfusion system at 40 min and lasting for 10 min (G post control) had no effect on the SR Ca^2+^ content of the cells isolated from the normal perfused heart. (D) The time-to-90% decay of caffeine-induced increases in [Ca^2+^]_i_ mainly reflect the Ca^2+^ clearance ability of the Na^+^/Ca^2+^ exchanger (NCX). n  =  18, 44, 44, 33, 38, 41 and 38 cells/3 mice in G post control, control, ischemia, G pre, G post, H pre and H post, respectively. (E) Representative western blots of the total phospholamban (PLB) and the phosphorylated PLB (p-PLB) in 6 groups and (F) the densitometric quantification of ratio of p-PLB/PLB (expressed as fold increase relative to control). n  =  5 mice in each group. Data are shown as means ± S.E.M. and analyzed by one-way ANOVA with *Tukey’s post hoc* test. *P < 0.05, **P < 0.01, *** *P* < 0.001 vs ischemic group.

A possible explanation for the decrease in the [Ca^2+^]_i_ transient amplitude (FIG. 3B and G) and the rate of increase in dR/d*t* of Ca^2+^ transients (FIG. 3E and J) after I/R may be a consequence of reductions in SR Ca^2+^ content [22], [26]. In order to estimate the SR Ca^2+^ content, we used caffeine (10 mM) to activate RyR2s and thoroughly deplete the SR, resulting in a significant rise in [Ca^2+^]_i_ (see the details in Methods and FIG. 4A [27]). The amplitude of caffeine-induced [Ca^2+^]_i_ transients and area under the transient curve can be used as estimates of the amount of Ca^2+^ stored in the SR [22]. Prior to caffeine application, cells were stimulated at 0.5Hz for 30s to normalize and replenish the SR Ca^2+^ content [22]. As shown in FIG.4B and C, there was a reduction in SR Ca^2+^ content after I/R that was restored by all GHS treatments.

To confirm whether GHS has an effect on the SR Ca^2+^ content of normal cardiomyocytes without ischemia, we performed ghrelin post-treatment on control hearts. We selected ghrelin post-treatment because it was the most effective at increasing SR Ca^2+^ content in I/R cardiomyocytes. The results (FIG. 4B and C) show no significant differences in area under the curve and amplitude of caffeine-induced Ca^2+^ transients between the control and ghrelin post-treated non-ischemic cardiomyocytes.

The time-to-90% decay of caffeine-induced Ca^2+^ transients mainly reflects the Ca^2+^ clearance ability of the NCX with a lesser contribution of sarcolemmal Ca^2+^ ATPase and mitochondrial uniporter [28], as the function of SERCA2a was counteracted by the opening of RyR2 in the presence of caffeine. As shown in FIG. 4D, the shortened time-to-90% decay of caffeine-induced Ca^2+^ transients after I/R indicates an accelerated Ca^2+^ clearance by the NCX. Therefore, this may suggest exaggerated NCX activity after I/R. GHS treatment prevented the reduction in the time-to-90% decay of caffeine-induced Ca^2+^ transients after I/R, which may suggest a normalization of NCX activity.

In order to further confirm whether these changes in the SR Ca^2+^ store were partially due to alterations in the uptake of Ca^2+^ into SR, the protein expression level of p-PLB and total PLB were examined by Western blotting (FIG. 4E). PLB and its phosphorylated form p-PLB have opposing effects on SERCA2a, as PLB inhibits SERCA2a activity and p-PLB releases this inhibition [28]. As shown in FIG. 4F, a significant decrease in p-PLB/PLB ratio after I/R was reversed by GHS treatment. This change corresponds with the recovery of SERCA2a activity after I/R by all GHS treatments. Taken together, these results further support our assertion that the protective effect of ghrelin on Ca^2+^ transients was achieved at least in part by the recovery of SERCA2a activity and SR Ca^2+^ content.

### GHS-R1a Mediates the Cardioprotective Effect of GHS

**Figure 5:**
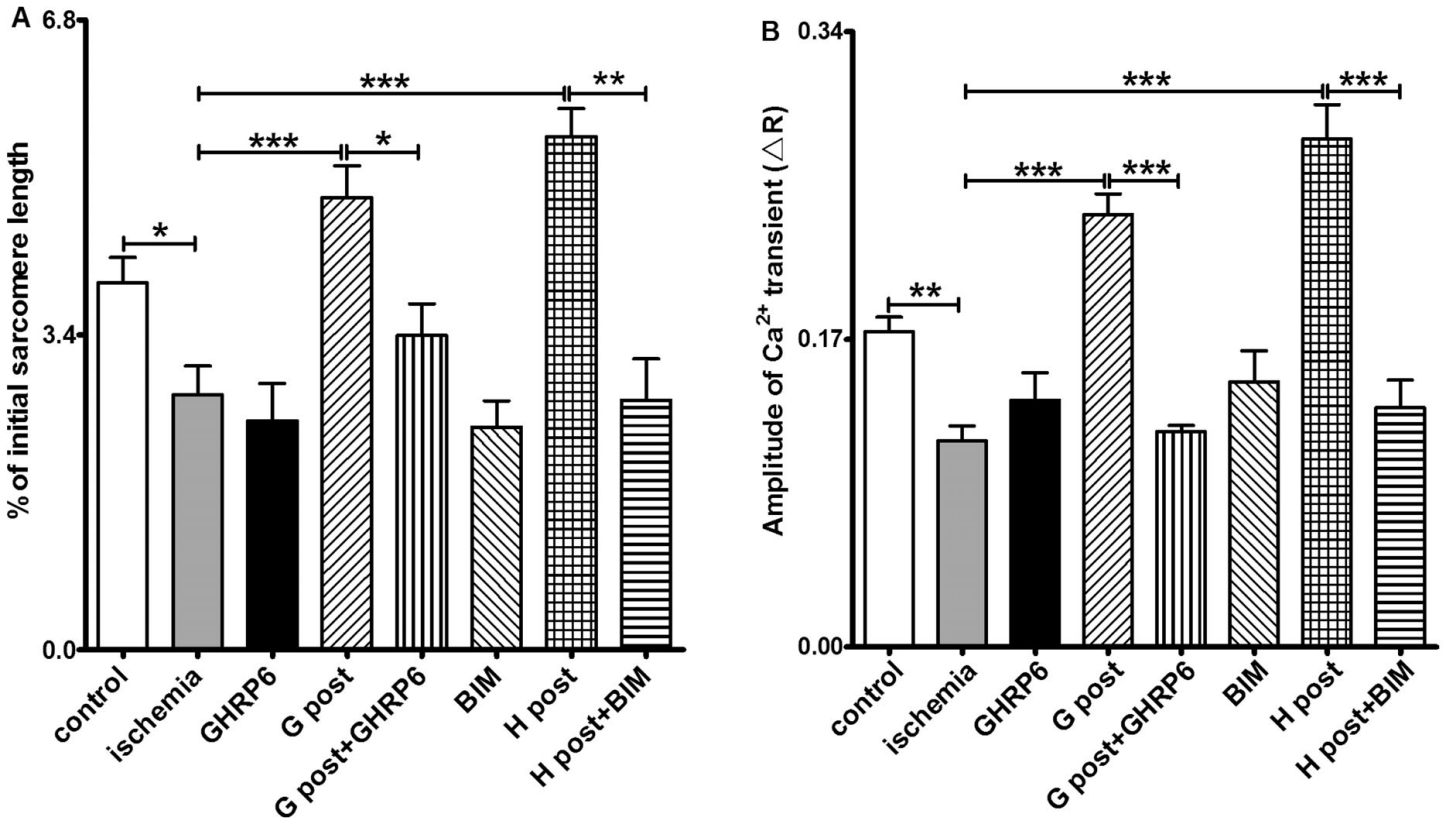
Role of GHS-R1a in the cardiac effects of hexarelin (H) and ghrelin (G). R represents the emission fluorescence ratio of fura-2 from excitation at 340 and 380nm. The GHS-R1a antagonists, [D-Lys3]-GHRP-6 (GHRP6, 200 nM) and BIM28163 (BIM,100 nM), completely blocked the effects of 1 nM hexarelin (H) and 10 nM ghrelin (G) post-treatment (post) on sarcomere shortening (A) and [Ca^2+^]_i_ transients (B). These peptides alone did not produce any noticeable change in sarcomere shortening (A) and [Ca^2+^]_i_ transients (B). For sarcomere shortening experiments, n  =  99, 84, 52, 95, 46,100, 50 and 61 in control, ischemic, GHRP6, G post, G post +GHRP6, BIM, H post and H post+BIM groups, respectively. For [Ca^2+^]_i_ transient experiments, n  =  72, 80, 55, 103, 69, 60, 66 and 68 in control, ischemic, GHRP6, G post, G post +GHRP6, BIM, H post and H post+BIM groups, respectively. Data were analyzed by one-way ANOVA with *Tukey's post hoc* test, and expressed as means ± S.E.M. *P < 0.05, ** *P* < 0.01, *** *P* < 0.001.

To investigate whether the GHS-induced effect on cardiomyocytes exposed to I/R injury was mediated by GHS receptors, we tested the GHS-R1a antagonists [D-Lys3]-GHRP-6 and BIM28163 [29], [30] in ghrelin and hexarelin post-treatment groups. [D- Lys3]-GHRP-6 (200 nM) or BIM28163 (100 nM) was introduced into the perfusion system 5 min before GHS post-treatment for a total of 15 min. Alone, neither of these antagonists showed any effect on the sarcomere shortening or [Ca^2+^]_i_ transients. However, both antagonists completely blocked the protective effects of ghrelin and hexarelin on sarcomere shortening (FIG. 5A) and [Ca^2+^]_i_ transients (FIG. 5B). These observations suggest that the protective effect of GHS is mediated by the GHS-R1a.

## Discussion

In the present study, we have confirmed that reduced cell shortening in mouse cardiomyocytes exposed to *in vitro* I/R injury is attributable to a reduction in both the amplitude and rising rate of [Ca^2+^]_i_ transients, which may be due to a reduced SR Ca^2+^ content caused by decreased SERCA2a activity. We have also demonstrated for the first time that GHS such as ghrelin and hexarelin, produce a protective effect on cardiomyocytes exposed to *in vitro* I/R injury. Normal cardiac myocyte contractility was maintained by a normal amplitude and rising rate of [Ca^2+^]_i_ transients after GHS treatment, which may be attributed to normalized SERCA2a activity and SR Ca^2+^content.

The mechanisms underlying cardiac ischemia are multifaceted, including ATP depletion, ROS generation, [Ca^2+^]_i_ overload amongst others. The disruption of Ca^2+^ homeostasis can cause inappropriate activation of Ca^2+^ dependent proteases and phospholipases essential for various cardiac functions, which may lead to further damage of the cardiomyocytes [2]. A tighter regulation of Ca^2+^ might therefore be an effective way to protect cardiomyocytes from I/R injury [6], [7].

As shown in our study, reduced heart function after I/R exists at the single cell level, as reflected by reduced cardiomyocyte contractility (FIG. 2) accompanied by a decrease in the [Ca^2+^]_i_ transient amplitude (FIG. 3) and an increase in the basal [Ca^2+^]_i_ (details in Results). In addition, the prolonged time for maximal sarcomere shortening after I/R (FIG. 2C and G) with unchanged time-to-peak Ca^2+^ transients (FIG. 3C and H) may reflect impairment of the contractile machinery essential for cell contraction, such as the degradation of the regulatory protein troponin [31]. Moreover, the reduction observed in the amplitude (FIG. 3B and G) and rising rate of [Ca^2+^]_i_ transients after I/R (FIG. 3E and J) could be due to decreases in voltage-gated L type Ca^2+^ current (*I*_CaL_) [32], SR Ca^2+^ content (FIG. 4B and C) or both. Any combination of these would reduce the magnitude of the SR Ca^2+^ release and subsequently alter the shortening phase of contraction. Our results certainly support the concept of a reduction in SR Ca^2+^ loading ability. This is shown by the reduced caffeine-induced Ca^2+^ transients, the reduction in the ratio of p-PLB to total PLB at the protein level and therefore the reduced activity of SERCA2a (FIG. 4). The reduced activity of SERCA2a is indicated by our data where we compared the time-to-90% decay of [Ca^2+^]_i_ transients under normal twitch with the time-to-90% decay of caffeine-induced Ca^2+^ transients. Under normal twitch, there was no significant difference in the time-to-90% decay of Ca^2+^ transients among experimental groups (FIG. 3D and I), however, the time-to-90% decay of caffeine-induced Ca^2+^ transients was shortened in ischemic group (FIG. 4D). Taken together, this suggests increased NCX activity but decreased SERCA2a activity after IR, with the decreased SERCA2a activity contributing to the decreased SR Ca^2+^ content. Nevertheless, the reduction in SR Ca^2+^ content may also be caused by an increase in the RyR2s-dependent and -independent Ca^2+^ leak from SR, as reported in cardiomyocytes from the failing rabbit heart [33].

A previous study from our laboratory has found that ghrelin and hexarelin exert a positive inotropic effect on normally perfused rat hearts [34] and isolated adult rat ventricular cardiomyocytes [21] through GHS-R1a [34], the reported functional receptor for GHS. This study also demonstrated that ghrelin and hexarelin pre- or post-treatments exert a protective effect on adult mouse ventricular cardiomyocytes during *in vitro* I/R injury via a positive inotropic effect and lusitropic effect (FIG. 2 and 3). Our results are consistent with those from previous studies using whole heart after I/R. Administrations of 20 nM ghrelin/1 µM hexarelin by Frascarelli et al. [17] or 0.1 nM-10 nM ghrelin by Chang et al. [18] protected the *in vitro* I/R rat heart from a reduction in cardiac function. Treatment with 320 µg/kg ghrelin or 80 µg/kg hexarelin (daily for 7 days) [10], or 10 nM/kg ghrelin (2 doses 12 h apart, the hearts were removed 1h after the last dose) [19] prevented I/R injury in the isolated rat heart by ameliorating the damaged heart function and attenuating the myocardial apoptosis. It has been suggested that ghrelin and hexarelin may exert their protective effects on rat heart I/R models through the activation of protein kinase C (PKC) [17] and/or inhibition of ER stress [19].

For the first time, we have demonstrated that the positive inotropic effect of GHS is at least partially due to the recovery or maintenance of normal SERCA2a activity (FIG. 4E and F) and therefore normal SR Ca^2+^ content (FIG. 4B and C). This is supported by the increased p-PLB/PLB protein ratio (FIG. 4F) after GHS treatment. Furthermore, when comparing the time-to-90% decay of [Ca^2+^]_i_ transients under normal twitch (FIG. 3D and I) and the time-to-90% decay of caffeine-induced Ca^2+^ transients (FIG. 4D), it suggests that SERCA2a activity was recovered after GHS treatment since the time-to-90% decay of caffeine-induced Ca^2+^ transients was restored back to control levels (normalization of NCX) by GHS treatment, whereas the time-to-90% decay of [Ca^2+^]_i_ transients under normal twitch had no significant change.

Ca^2+^ overload, which has been reported as the main damaging factor causing cardiac dysfunction after ischemia [2], was also observed in this study (see results for details). The basal level of [Ca^2+^]_i_ was increased in ischemic cells with or without GHS treatment. The basal level of [Ca^2+^]_i_ would reflect the activity of both SERCA2a and RyR2s [35]. Following the recovery of SERCA2a activity by GHS treatment (FIG. 4F), SR Ca^2+^ content was also restored. If the sensitivity of RyR2s to Ca^2+^ is changed in ischemia (Ca^2+^ leak from SR), increased Ca^2+^ leak from SR may occur. Such a presumption seems consistent with an even greater increase in the basal level of [Ca^2+^]_i_ after GHS treatment. Moreover, the increased basal [Ca^2+^]_i_ in GHS treated groups may also be due to the increased Ca^2+^ influx into the cell through *I*_CaL_,which has been reported previously by our group [21]. To what extent this effect contributes to the improvements in Ca^2+^ homeostasis and cardiomyocyte function seen in the current study requires further investigation.

We show here the expression of GHS-R1a in the mouse heart at both the mRNA and protein level (FIG.1). In order to determine whether the protective effect of GHS on cardiomyocytes after I/R injury was mediated by GHS-R1a, we administrated the GHS-R1a antagonists [D-Lys3]-GHRP-6 or BIM28163 along with GHS in I/R hearts. There is no report of non-specific effects of BIM28163 and [D-Lys3]-GHRP-6, and both reagents are widely applied as selective GHS-R1a antagonists in different cell types (eg neurons) [36], organs (stomach, small intestine) [37], [38], [39], and species (rodents [36], [38], dogs [37]). The GHS-R1a antagonists alone showed no effect on cardiomyocytes, but completely blocked the effects of ghrelin or hexarelin on sarcomere shortening and [Ca^2+^]_i_ transients of cardiomyocytes after I/R injury (FIG. 5). This suggests that the effects of GHS, especially their actions on the contractile properties of cardiomyocytes and the corresponding increases in [Ca^2+^]_i_ transients, are mediated by GHS-R1a.

In summary, ghrelin and hexarelin pre- and post-treatments protected mouse cardiomyocytes from *in vitro* I/R injury and preserved the cell shortening by regulating [Ca^2+^]_i_. Both ghrelin and hexarelin prevented the decrease in the amplitude of [Ca^2+^]_i_ transients and SR Ca^2+^ content after I/R by maintaining the ratio of p-PLB to total PLB and therefore the SERCA2a activity. Their positive inotropic effect on the cardiomyocytes and corresponding increase in [Ca^2+^]_i_ transients is mainly mediated by the activation of GHS-R1a.
